# Virtual reality, real-world rhythm: evaluating gamified training for medical students

**DOI:** 10.3389/fpubh.2026.1833908

**Published:** 2026-08-03

**Authors:** Florian Ettl, Felix Eibensteiner, Robert Greif, Stefan Mayhardt, Stefan Nern, Luca Michael Kurz, Benedict Alan Daniels, Günther F. Körmöczi, Anahit Anvari-Pirsch, Jürgen Grafeneder, Christoph Schriefl

**Affiliations:** 1 Department of Emergency Medicine, Medical University of Vienna, Vienna, Austria; 2 Faculty of Medicine, University of Bern, Bern, Switzerland; 3 Department of Transfusion Medicine and Cell Therapy, Medical University of Vienna, Vienna, Austria; 4 Teaching Center, Medical University of Vienna, Vienna, Austria

**Keywords:** advanced life support, gamification, medical education, OSCE, resuscitation, resuscitation training, virtual reality

## Abstract

**Background:**

Virtual reality (VR) and gamification are increasingly recommended as adjuncts in resuscitation education, but their integration into undergraduate medical curricula and data on usability and learner acceptance remain limited. We evaluated a newly developed, gamified VR training application for medical students during their preparation for an Objective Structured Clinical Examination (OSCE).

**Methods:**

In this single-centre observational questionnaire study, fourth-year medical students completed VR-based advanced life support (ALS) training with randomized electrocardiogram (ECG) rhythms and gamified decision tasks. The primary outcome was usability measured with the Virtual Reality System Usability Questionnaire (VRSUQ; 0–100 points). Secondary outcomes included VR-induced symptoms (VRSQ; 0–100 points), self-rated rhythm-analysis competence before and after training (1–10), self-rated in-app performance (0–10), and trainer-reported feasibility. Continuous variables are presented as median (interquartile range [IQR]). Associations between outcomes were assessed using Spearman rank correlation coefficients.

**Results:**

Of 660 eligible students, 294 (44.5%) participated in the VR-study and completed the questionnaires. Usability was high (VRSUQ 83.3 [IQR 72.2–91.7]). VR-induced symptoms were low (VRSQ 8.3 [IQR 0.0–16.7]). Self-rated competence increased from 5.0 (IQR 4.0–7.0) before training to 8.0 (IQR 7.0–8.0) after training (*p* < 0.001), corresponding to a competence gain of 2.0 points (IQR 1.0–3.0). Self-rated performance was 7.0 (IQR 5.0–8.0) and correlated with usability (*ρ* = 0.44, *p* < 0.001). Overall training quality was rated 9.0 (IQR 8.0–10.0), and 91.9% would participate again. Usability did not differ between genders but was higher among students with prior VR experience. Trainers reported short setup times, manageable supervision ratios, and occasional technical support needs.

**Conclusion:**

Gamified VR-based ALS training for medical students is feasible, demonstrates high usability and low symptom burden, and improves self-assessed competence. VR appears to be a well-accepted adjunct to undergraduate resuscitation education.

## 1 Introduction

Cardiac arrest remains a major global public health issue, surpassing any individual cancer in premature mortality and accounting for nearly half of all cardiovascular deaths (1). Due to the low survival rates ranging from 2–11%, the importance of early intervention in cardiac arrest cannot be overstated (2). Resuscitation training of health care professionals is associated with improved outcomes after cardiac arrest, increased rates of return of spontaneous circulation, and improved survival to hospital discharge and at 30 days (3–7). Thus, effective cardiopulmonary resuscitation (CPR) training is crucial for skill acquisition, for retention of competences, and finally for survival of patients in cardiac arrest. Traditional CPR training methods have shown poor skill retention after 6 months (8). In contrast, low-dose, high-frequency training programs have demonstrated improved CPR skill retention (9–14). Low-dose, high-frequency (LDHF) training consists of brief, repetitive practice sessions distributed over time rather than concentrated in a single intensive training period. This approach has been associated with improved skill retention and reduced skill decay (13, 14). In this context, high-quality training is characterized by standardized, feedback-rich practice focused on specific skill components (15, 16). Virtual reality is well suited to facilitate LDHF training by providing standardized scenarios, immediate objective feedback, and unlimited opportunities for repetition (17, 18). Once established, VR training is readily accessible, can be undertaken at any time with minimal logistical effort, and requires few personnel or space resources, enabling scalable self-directed practice.

Evidence has shown that personnel employed within emergency departments and intensive care units who engage in regular CPR practice demonstrate enhanced skills retention (19). Furthermore, the quality of training also plays a vital role (20). Instructor-led training has been shown to be equal to a self-learner station in skill retention (21, 22).

### 1.1 Virtual reality in CPR training

Virtual Reality (VR) has emerged as an innovative CPR training method, offering immersive, interactive experiences that potentially improve learning and skill retention. The effectiveness of virtual reality basic life support (BLS) training in improving CPR quality has been demonstrated, with particular benefits observed for lay rescuers; however, its effect is controversial in the current literature (6, 23–25).

That potential of VR in CPR training has even been recognised by the International Liaison Committee on Resuscitation (ILCOR) in the 2024 International Consensus on Cardiopulmonary Resuscitation and Emergency Cardiovascular Care Science with Treatment Recommendations (CoSTR) suggesting that VR can be considered an alternative or supplement to traditional CPR training methods with the potential to improve skill acquisition and retention (6). The evolution of training methods from traditional approaches to innovative technologies presents opportunities to improve skill acquisition and retention while potentially increasing learners’ motivation by offering more engaging and immersive experiences (26). The integration of VR and gamification into CPR training reflects broader technology-enhanced learning trends in medical education and is included in current teaching recommendations for resuscitation (27, 28). Gamification describes the integration of game-like elements - such as competitions, point systems, progressive levels of difficulty, and leaderboards into educational settings, most commonly through digital formats, to promote interactive and intuitive learner engagement (29). In resuscitation education, gamified approaches have been shown to enhance learners’ knowledge, skills, attitudes, and engaged participation, particularly with respect to teamwork and problem-solving (27, 29, 30).

Despite growing interest in VR-based medical education, several questions remain unanswered. First, the usability of VR applications in real-world educational settings requires systematic evaluation using standardized assessment instruments. Usability is defined as the extent to which a system can be used by specified users to achieve specified goals with effectiveness, efficiency, and satisfaction (31). In educational contexts, high usability is essential to minimize extraneous cognitive load and ensure that learners can focus on the educational content rather than system operation (32).

Second, VR-induced discomfort remains an important consideration. It arises from sensory conflict between visual and vestibular inputs and may manifest as nausea, dizziness, eye strain, and related symptoms. Assessing these effects is critical, as discomfort may reduce learner acceptance, impair training effectiveness, and limit repeated use of VR-based training (33).

Third, the impact of VR training on learners’ self-assessed competence warrants further investigation. Self-assessed competence reflects an individual’s perceived ability to perform a specific task and has been associated with motivation to practice, willingness to engage in clinical situations, and subsequent learning behaviour (34, 35). While self-assessment cannot be considered a direct measure of actual competence or performance, it provides valuable insights into learners’ perceived preparedness and confidence to act in real clinical emergencies (36).

To harness the potential of virtual reality in teaching skills needed to treat patients in cardiac arrest, a VR application was specifically developed to prepare students for the Objective Structured Clinical Examination (OSCE) at the Medical University of Vienna, aiming to help medical students to learn and consolidate the theoretical principles of rhythm analysis within the Advanced Life Support (ALS) framework through an interactive, gamified experience. Reinforcing that conceptual knowledge should improve students’ readiness for hands-on ALS training and their performance in the OSCE.

This questionnaire study aims to evaluate medical students’ perceptions of the gamified VR training, specifically on its educational value and usability. Additionally, we recorded the technical VR performance, user-reported issues like virtual reality-induced symptoms, and insights from instructors in that training experience.

## 2 Methods

### 2.1 Study design

This observational study evaluated the quality of a newly developed VR-based, gamified application for rhythm analysis embedded in a resuscitation scenario, as well as the user experience based on a questionnaire.

#### 2.1.1 VR-based gamified rhythm analysis application

The VR application was developed for the Pico 4 headset (ByteDance/Pico, Mountain View, California, United States) and begins by explaining the training context and the participant’s tasks to perform. Users could then choose between a guided tutorial with a brief introduction to the VR environment and its functions, or a direct start to the rhythm analysis training scenario. The tutorial included step-by-step instructions, with an audio guide explaining how to interact with the VR application, allowing users to familiarise themselves with the controls and procedures.

The gamified scenario offered three levels of difficulty: (1) standard, (2) advanced, and (3) expert. Standard mode, recommended for first-time users after completion of the tutorial, allowed unlimited time for rhythm recognition and execution of the appropriate clinical actions. In advanced mode, participants were required to complete these tasks within 28 s for each ECG rhythm, whereas expert mode reduced the time limit to 22 s and added visual and auditory stressors to increase the challenge and provide more situational learning. Participants were free to select, switch between, and repeat difficulty levels throughout the training session. Difficulty levels were part of the structured training design rather than separate study groups. Accordingly, no random allocation was performed.

**Figure 1:**
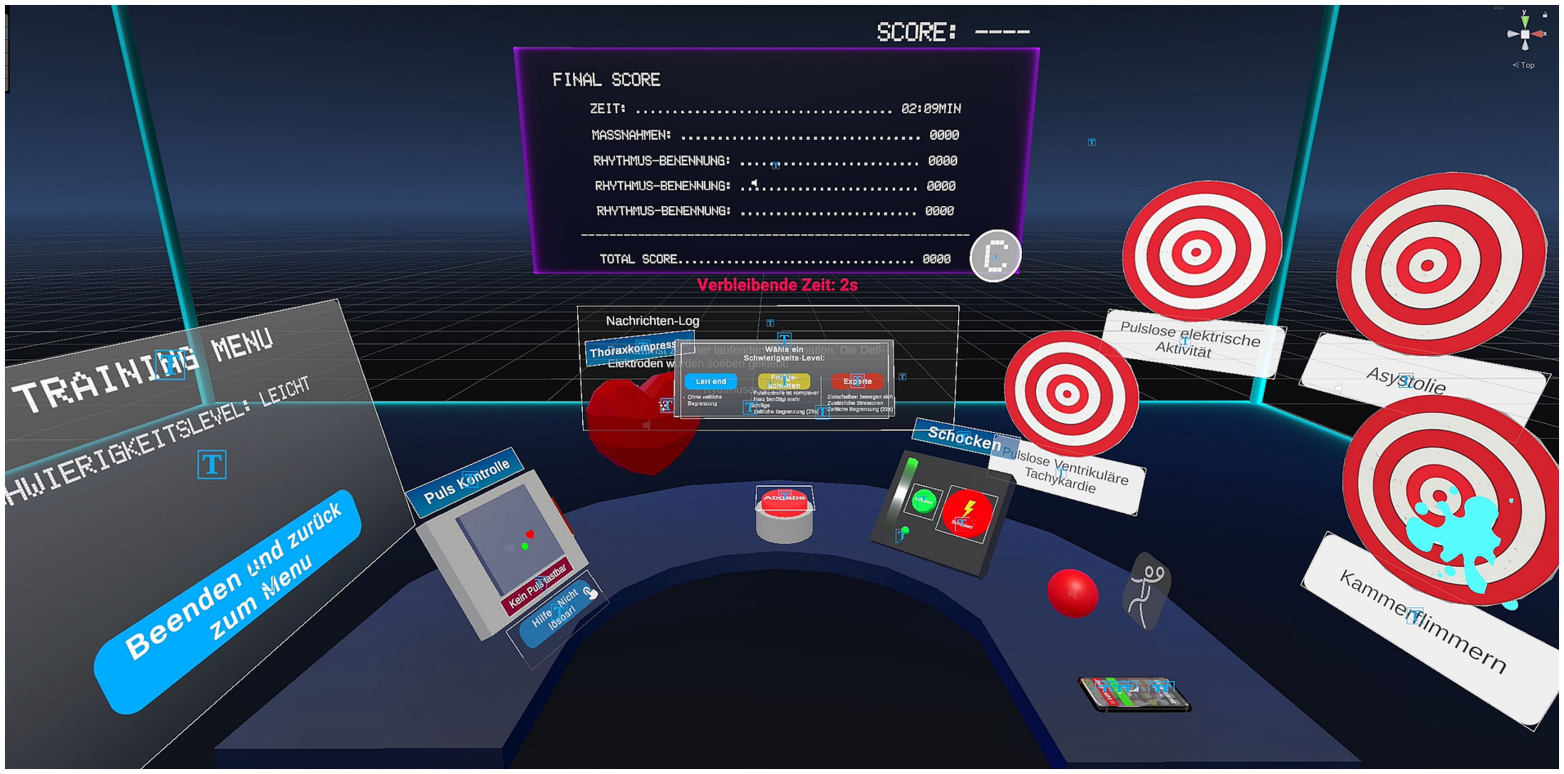
Visual example of the VR interface and gameplay.

**Figure 2:**
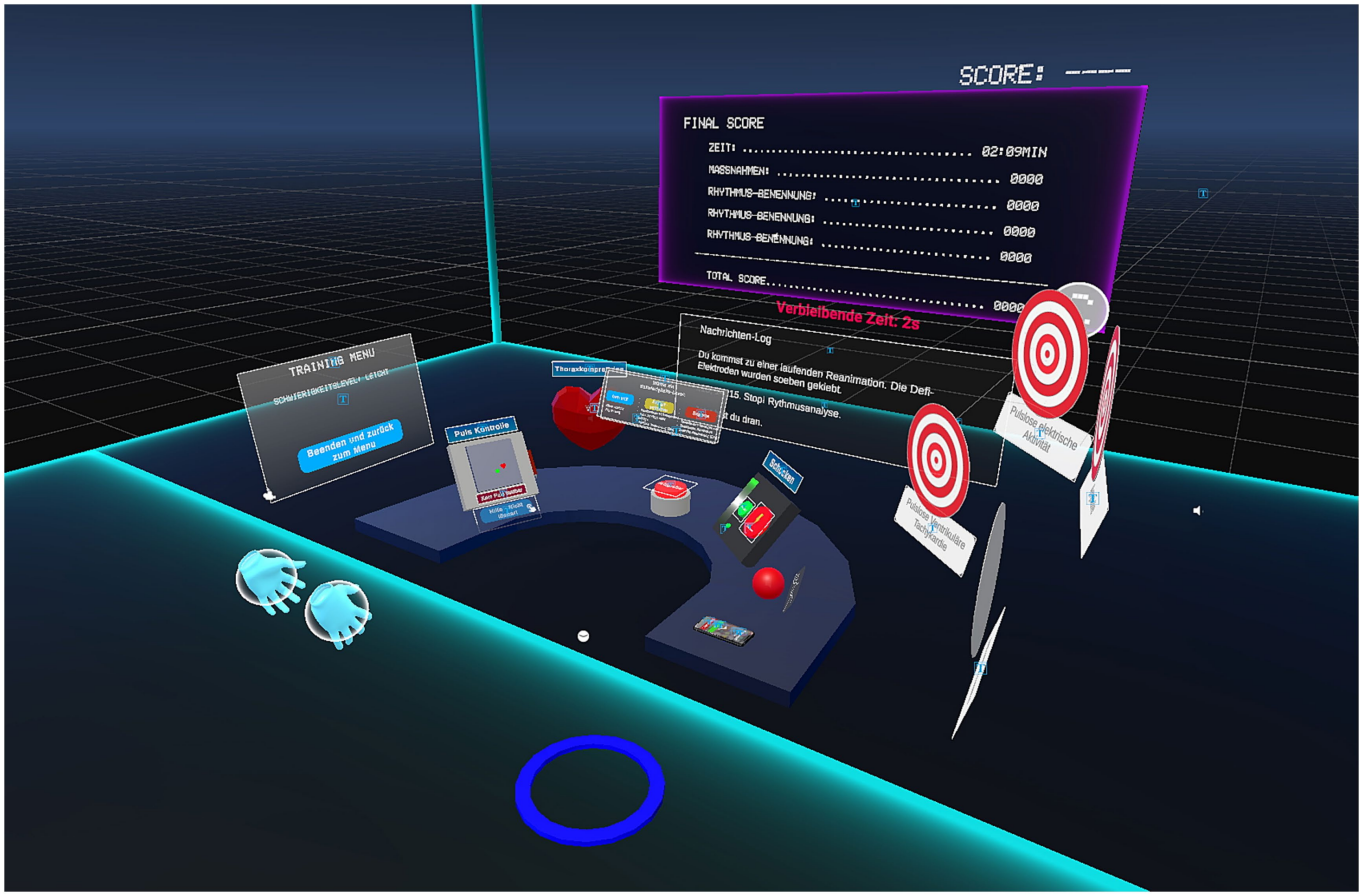
Visual example of the VR interface and gameplay.

After starting a rhythm training, electrocardiogram (ECG) rhythms were drawn from a predefined pool and presented in random order for each session. A total of 20 rhythm examples were available; from these, 12 were randomly selected. Participants were required to interpret the rhythms, select the appropriate immediate action (pulse check, defibrillation, or chest compressions), and correctly identify the rhythm (ventricular fibrillation, pulseless ventricular tachycardia, asystole, or pulseless electrical activity) as quickly and accurately as possible. Responses were implemented through gamified VR interactions, such as activating a virtual defibrillator lever, performing chest compressions by pressing a heart-shaped virtual sensor with both hands, and selecting rhythm labels by throwing a virtual paint bag at the corresponding target. The system provided immediate corrective feedback on both rhythm classification and treatment decisions. Upon completion, participants received an individual performance summary listing correct and incorrect decisions together with the correct responses. Competitive scoring elements were included to enhance learner motivation. Performance was scored based on accuracy and speed. Visual examples of the VR interface and gameplay are shown in Figures 1, 2.

### 2.2 Study setting

The study was conducted at the Teaching Centre of the Medical University of Vienna as a voluntary training session preparing the students for the mandatory OSCE in the year four of the study of human medicine. VR training was integrated into the existing three-week OSCE preparation period, during which students also participated in conventional instructor-led ALS training as well as other OSCE-related learning activities, including ECG interpretation, communication skills training, procedural skills practice, and independent study. The target population comprised approximately 660 fourth-year medical students. During these training sessions, a VR experienced and ALS certified trainer provided the virtual reality experience and supervised participating students in a specific study room with six VR headsets having six training positions, each measuring 2 × 2 metres.

All fourth-year medical students who were able and willing to provide oral and written consent were included. As the study was exploratory in nature and focused on feasibility and user experience rather than hypothesis testing of a predefined effect size, no formal sample size calculation was conducted. The target population was defined as all eligible fourth-year medical students (*n* ≈ 660) enrolled during the 3-week OSCE preparation period. Based on previous experience with voluntary educational activities, a participation rate of approximately 50% was anticipated. Therefore, a pragmatic convenience sample was used. Given the lack of prior data on the investigated VR application and the exploratory nature of the study, a formal *a priori* sample size calculation was not considered feasible. Students with known motion sickness or a history of known epilepsy or seizures were excluded. After inclusion and completion of the VR scenario, participants completed a questionnaire comprising three major parts (Appendix A). The first part included questions about the participants’ characteristics and their self-rated competence in handling a rhythm analysis before and after the training (on a 10-point Likert scale). The second was the standardised “Virtual Reality System Usability Questionnaire” (VRSUQ) (31), based on the system usability model focusing on users’ experience. The third part determined motion sickness possibly suffered in the virtual reality environment using the standardised “Virtual Reality Sickness Questionnaire” (VRSQ) (37). Both the pre-training and post-training competence ratings were collected after completion of the VR session. However, as the training sessions were relatively short, we assumed that a recall bias would be negligible.

Each trainer completed a questionnaire for each half-day (5 h), covering organisational and technical aspects, trainer satisfaction, and a subjective assessment of the student’s motivation on a 10-point Likert scale. The questionnaire with 14 different questions is provided in Appendix B.

This study complies with the Declaration of Helsinki and was reviewed by the Ethics Committee of the Medical University of Vienna. After review, an exemption from formal ethical approval was granted. Following the Ethics Committee’s guidance and institutional requirements, the study was reviewed and approved by the Data Protection Committee and the Teaching Review Board of the Medical University of Vienna.

The present study aimed to evaluate the usability and learner acceptance of a newly developed gamified VR application for rhythm analysis in undergraduate ALS training. In addition, we sought to assess VR-induced symptoms, self-rated competence before and after training, and trainer-reported feasibility. We hypothesized that the application would be perceived as usable, with low levels of VR-induced symptoms and higher self-rated competence after training. The study addressed the following research questions: (1) How usable is the newly developed gamified VR rhythm analysis application from the learners’ perspective? (2) To what extent do learners experience VR-related symptoms during training? (3) How do learners rate their competence in rhythm analysis before and after the VR training? and (4) How feasible is the implementation of the application from the trainers’ perspective?

### 2.3 Objectives

The primary objective of this study was the user experience assessed by the Virtual Reality System Usability Questionnaire (VRSUQ). The VRSUQ is a validated nine-item instrument specifically designed for virtual reality systems and covers effectiveness, efficiency, and user satisfaction (31). Items are rated on a 5-point Likert scale from 1 (strongly disagree) to 5 (strongly agree). Negatively worded items (errors, cybersickness symptoms, and mental stress) are reverse-coded so that higher values indicate better usability. The overall usability score is calculated as the mean of all items and transformed to a 0–100 scale using the formula (mean − 1) × 25, with higher scores representing greater perceived usability, as originally validated. This standardized transformation facilitates interpretation and comparability of results while preserving the relative ranking of participants. The full questionnaire is provided in Appendix A.

Secondary objectives included the self-rated assessment of students’ competence in rhythm analysis before and after completing the VR training, the correlation between the students’ self-assessed performance in the application and the VRSUQ usability score, and the perceived sickness during the application of the scenario using the standardised Virtual Reality Sickness Questionnaire (VRSQ, 6 questions with 4 points from 1 [none] to 4 [severe]) (37). VRSQ scores were likewise transformed to a standardized 0–100 scale according to the original scoring procedure, with higher scores indicating more severe VR-related symptoms. Furthermore, the VR trainers’ teaching and support experience with the VR goggles’ usability and the scenario was assessed using a 14-item questionnaire (Appendix B).

#### 2.3.1 Statistical analysis

Continuous variables are presented as median and interquartile range (IQR; 25th–75th percentile) and categorical variables as counts and percentages. Histograms were generated to visually assess score distributions (Appendix C).

Within-participant changes in self-rated competence before and after VR training were assessed using the Wilcoxon signed-rank test. Between-group comparisons were performed using the Mann–Whitney *U* test.

Associations between questionnaire outcomes were assessed using Spearman rank correlation coefficients.

All statistical analyses were performed using R version 4.3.0 (“Already Tomorrow” (38). Data management and statistical analyses were conducted using the dplyr (39) and gtsummary (40) packages, while tables and figures were generated using flextable (41), officer (42), and ggplot2 (43). A two-sided *p*-value < 0.05 was considered statistically significant.

## 3 Results

### 3.1 Student questionnaire

#### 3.1.1 Baseline

**Figure 3:**
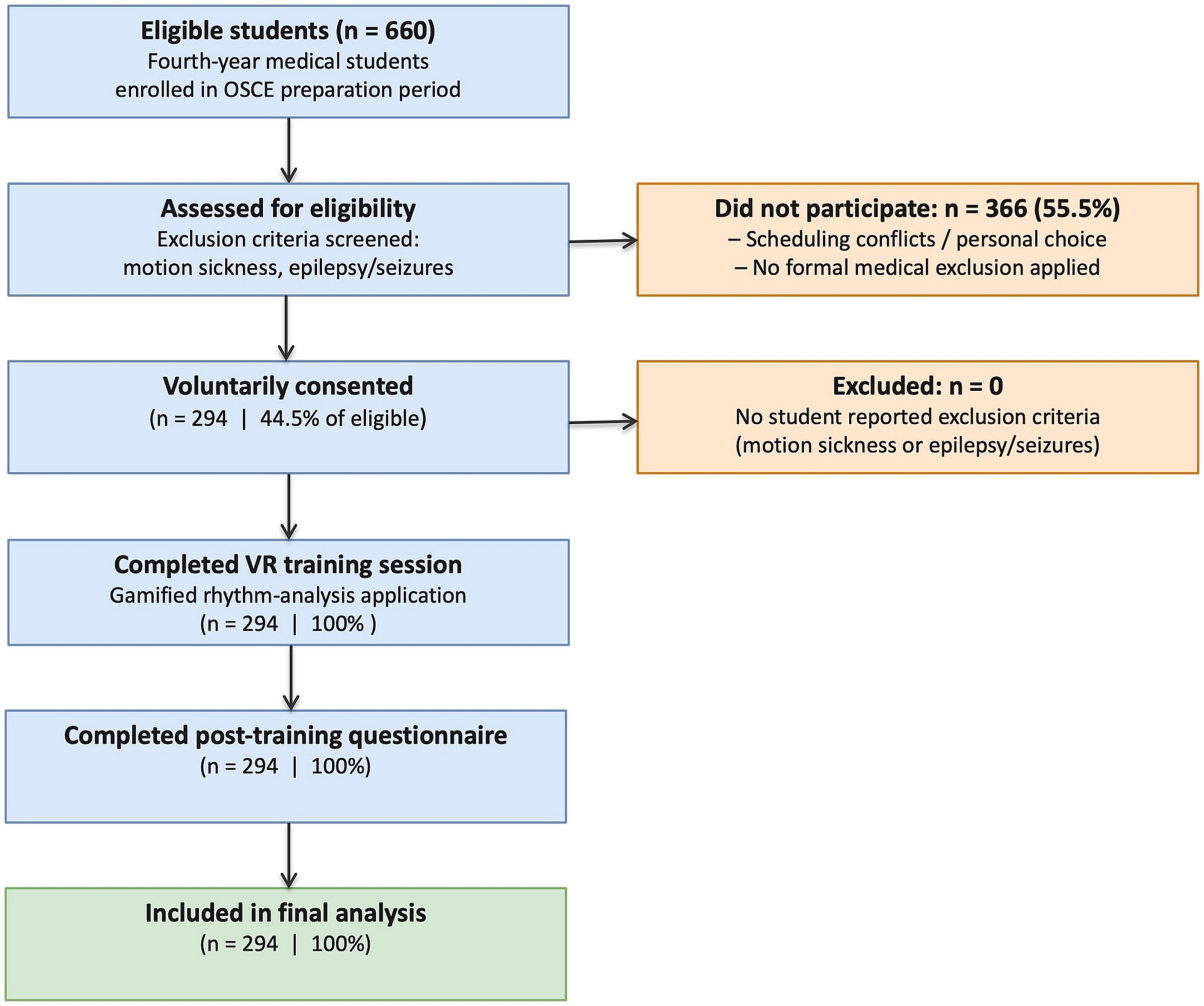
Participant flow diagram.

Of 660 eligible medical students, a total of 294 (44.5%) participated in the VR rhythm-analysis training and completed the post-training questionnaire. The remaining 366 students (55.5%) did not participate, predominantly due to scheduling conflicts and personal choice not to engage in the voluntary session; no student was formally excluded on medical grounds, as no volunteering participant reported a history of motion sickness or epilepsy or seizures. The complete participant flow from eligibility screening to final analysis is illustrated in Figure 3. Age distribution was relatively homogeneous, and gender was balanced (female 50.0%, male 49.3%, diverse 0.3%). Participant’s age was recorded because age may influence familiarity with digital technologies, acceptance of VR-based training, and susceptibility to VR-related symptoms. Participants’ characteristics are presented in Table 1.

**Table 1:** Sample characteristics of the study population (*n* = 294).

Characteristic	Category	*n* (%)
Gender	Male	145 (49.3)
	Female	147 (50.0)
	Diverse	1 (0.3)
	Not reported	1 (0.3)
Age (years)	21–23	131 (44.6)
	24–26	124 (42.2)
	27–29	25 (8.5)
	≥30	14 (4.7)
Prior medical qualification^*^	Any	104 (35.4)
Prior experience with VR	Yes	238 (81.0)

Data are presented as *n* (%). Percentages may not sum to 100 due to rounding. VR, virtual reality. ^*^Prior medical qualification: nurse or paramedic.

Of 660 eligible fourth-year medical students, 294 (44.5%) voluntarily participated in the VR rhythm-analysis training and completed the post-training questionnaire. The remaining 366 students (55.5%) did not participate, predominantly due to scheduling conflicts and personal choice not to engage in the voluntary session; no student was formally excluded on medical grounds, as no volunteering participant reported a history of motion sickness or epilepsy. The complete participant flow from eligibility screening to final analysis is illustrated in Figure 3.

#### 3.1.2 Endpoints

The primary endpoint, usability assessed by the VRSUQ (0–100), was high, with a median score of 83.3 (IQR 72.2–91.7). VR-induced symptoms measured by the VRSQ (0–100) were generally low, with a median score of 8.3 (IQR 0.0–16.7).

Self-rated competence increased from 5.0 (IQR 4.0–7.0) before training to 8.0 (IQR 7.0–8.0) after training (Wilcoxon signed-rank test, *p* < 0.001), corresponding to a median competence gain of 2.0 points (IQR 1.0–3.0). Detailed pre- and post-training competence ratings are presented in Table 2. Self-rated performance within the VR scenario was 7.0 (IQR 5.0–8.0).

**Table 2:** Self-rated competence before and after VR training.

Group	Competence before VR	Competence after VR	Competence change
Male	6.0 (5.0–8.0)	8.0 (7.0–9.0)	1.0 (1.0–2.0)
Female	5.0 (3.0–7.0)	8.0 (6.0–8.0)	2.0 (1.0–3.0)
*p*-value	<0.001	0.061	0.002
Prior medical qualification	7.0 (5.0–8.0)	8.0 (7.0–9.0)	1.0 (0.0–2.0)
No prior medical qualification	5.0 (4.0–7.0)	7.0 (6.0–8.0)	2.0 (1.0–3.0)
*p*-value	<0.001	0.001	<0.001
Prior VR experience	6.0 (4.0–7.0)	8.0 (7.0–9.0)	2.0 (1.0–3.0)
No prior VR experience	5.0 (3.0–6.0)	7.0 (5.0–8.0)	2.0 (0.0–3.0)
*p*-value	0.014	<0.001	0.639

Data are presented as median (interquartile range). *p*-values were calculated using the Mann–Whitney *U* test.

Participants reporting diverse or not reported gender were excluded from gender subgroup analyses (*n* = 2).

Usability showed a moderate positive correlation with self-rated performance (Spearman’s *ρ* = 0.44, *p* < 0.001). A moderate negative correlation was observed between VRSQ and VRSUQ scores (Spearman’s *ρ* = −0.40, *p* < 0.001), indicating that participants reporting more VR-related symptoms tended to rate the usability of the application lower.

Although eye strain (45.6%) and blurred vision (42.2%) were frequently reported, symptom severity was generally low, and most ratings fell within the mild range. No participant discontinued the VR session because of VR-related discomfort. VR-related symptoms were not associated with competence gain (Spearman’s *ρ* = 0.05, *p* = 0.395) and were not associated with willingness to participate in future VR training (Spearman’s ρ = −0.03, *p* = 0.634).

Overall training quality was rated 9.0 (IQR 8.0–10.0). Regarding training preferences, 120 students (40.8%) preferred VR-based training, whereas 174 (59.2%) preferred traditional training (*p* < 0.001). Despite this preference, most participants indicated that they would participate in VR training again (*n* = 271, 91.8%). Preferred settings for future VR training included university training rooms (58.8%), integration into mandatory curriculum components (38.8%), elective courses (29.9%), and home-based use (14.3%).

#### 3.1.3 Subgroup analyses

Subgroup analyses are presented in Table 3. Participants reporting diverse or not reported gender (*n* = 2) were excluded from gender subgroup analyses.

**Table 3:** Usability, VR-induced symptoms, competence change, and performance outcomes stratified by participant characteristics.

Group	Usability (VRSUQ score)	VR-induced symptoms (VRSQ score)	Competence change	Performance
Male	83.3 (75.0–88.9)	4.2 (0.0–12.5)	1.0 (1.0–2.0)	7.0 (6.0–8.0)
Female	83.3 (72.2–91.7)	8.3 (4.2–25.0)	2.0 (1.0–3.0)	6.0 (5.0–8.0)
*p*-value	0.592	<0.001	0.002	0.072
Prior medical qualification	83.3 (72.2–88.9)	4.2 (0.0–16.7)	1.0 (0.0–2.0)	7.0 (5.0–8.0)
No prior medical qualification	83.3 (72.2–91.7)	8.3 (4.2–20.8)	2.0 (1.0–3.0)	7.0 (5.0–8.0)
*p*-value	0.330	0.010	<0.001	0.074
Prior VR experience	86.1 (75.0–91.7)	8.3 (0.0–20.8)	2.0 (1.0–3.0)	7.0 (5.0–8.0)
No prior VR experience	77.8 (63.9–86.1)	8.3 (0.0–12.5)	2.0 (0.0–3.0)	6.0 (5.0–8.0)
*p*-value	0.003	0.709	0.639	0.019

Data are presented as median (interquartile range). *p*-values were calculated using the Mann–Whitney *U* test.

VRSUQ, Virtual Reality Simulation Usability Questionnaire; VRSQ, Virtual Reality Sickness Questionnaire.

Higher VRSUQ scores indicate better usability, whereas higher VRSQ scores indicate more severe VR-induced symptoms.

Participants reporting diverse or not reported gender were excluded from gender subgroup analyses (*n* = 2).

Usability did not differ between male and female participants (83.3 [75.0–88.9] vs. 83.3 [72.2–91.7]; *p* = 0.592). Female participants reported significantly higher VRSQ scores (8.3 [4.2–25.0] vs. 4.2 [0.0–12.5]; *p* < 0.001). Male participants reported higher pre-training competence ratings than female participants (6.0 [4.0–7.0] vs. 5.0 [4.0–7.0]; *p* = 0.027), whereas post-training competence ratings were comparable between groups (8.0 [7.0–8.0] vs. 8.0 [7.0–8.0]; *p* = 0.653). Consequently, female participants demonstrated a greater competence gain (2.0 [1.0–3.0] vs. 1.0 [1.0–2.0]; *p* = 0.002). Self-rated performance within the VR scenario did not differ significantly between groups (*p* = 0.072).

Participants without prior medical qualification reported greater competence gain (2.0 [1.0–3.0] vs. 1.0 [0.0–2.0]; *p* < 0.001), whereas those with prior medical qualification reported lower VRSQ scores (4.2 [0.0–16.7] vs. 8.3 [4.2–20.8]; *p* = 0.010). Usability and self-rated performance did not differ significantly between groups.

Participants with prior VR experience reported higher usability scores (86.1 [75.0–91.7] vs. 77.8 [63.9–86.1]; *p* = 0.003) and higher self-rated performance (7.0 [5.0–8.0] vs. 6.0 [5.0–8.0]; *p* = 0.019) than participants without prior VR experience. VRSQ scores and competence gain were comparable between groups.

### 3.2 Trainer questionnaire

Across the study period, four trainers completed 28 trainer questionnaires. Median preparation time at the beginning of each half-day session (5 h) was 10 min (IQR 8–15 min). Trainers supervised 4–6 students simultaneously in 60.7% of sessions, 1–3 students in 28.6%, 7–9 students in 7.1%, and no students in 3.6%.

During 5-h sessions, 1–3 technical issues occurred in 60.7% of cases. Support was required 1–3 times in 78.6% of sessions for headset handling highly, with a median score of 8.0 (IQR 7.0–9.0) on a 10-point Likert scale.

## 4 Discussion

This observational study evaluated medical students’ perceptions of a VR-based, gamified rhythm-analysis teaching integrated into ALS preparation. The main findings demonstrate high usability ratings (VRSUQ 83/100), minimal VR-induced symptoms (VRSQ 8/100), and significant self-assessed competence increase (from 5.0 to 8.0/10), suggesting that VR-based rhythm-analysis training is feasible, well-accepted, and educationally valuable within undergraduate resuscitation education.

The primary outcome revealed a highly accepted usability measured by the VRSUQ (83/100), which is consistent with previous VR-based procedural trainings reporting a median System Usability Scale score of about 83 (32, 44). High usability is critical for successful curricular integration, as suboptimal interface design may impair learning outcomes (45). In VR-based medical education, poor usability may increase extraneous cognitive load, divert attention from the learning task, and reduce acceptance of the technology. Conversely, intuitive and user-friendly systems allow learners to focus on educational content and may facilitate repeated engagement with training, which is considered important for skill acquisition and retention in resuscitation education (32, 46). The moderate positive correlation between usability and self-rated performance (Spearman’s *ρ* = 0.44, *p* < 0.001) suggests that intuitive VR environments enhance learners’ perceived effectiveness by allowing them to focus on educational content rather than system operation (32, 46).

The significant increase in self-rated competence with a change of about 2.0 points indicates substantial improvement in students’ confidence. Self-rated competence, as a proxy for self-efficacy, is a recognised outcome measure in simulation-based medical education, as it reflects learners’ perceived readiness to act and has been associated with quality of clinical care and willingness to engage in clinical situations (47). Although self-rated competence does not directly measure objective performance, it captures motivational and behavioural dimensions relevant to emergency response readiness. A small-scale study showed that VR training offers advantages over traditional training methods, particularly in theoretical knowledge and practical application (48). A recent randomized controlled trial demonstrated that VR-based training can serve as an effective alternative for traditional training, with particular benefits for knowledge acquisition (49). The high training quality rating of 9.0/10 and that over 90% of students would participate again in VR-training underscore educational value and acceptability of that intervention. Our findings contribute to the growing body of evidence supporting VR in resuscitation education. Our focus on rhythm analysis-a cognitive rather than psychomotor skill-may explain the particularly favourable outcomes.

The mean VRSQ score of 8/100 indicates a low overall burden of VR-related symptoms. Item-level analyses showed that the most frequently reported symptoms were generally mild, and no participant discontinued the VR session because of VR-related discomfort. Although this value appears lower than the pooled Simulator Sickness Questionnaire rating published in a systematic review (50), it should not be directly compared, as the two instruments differ in item content and scoring. Female participants reported significantly higher VRSQ scores, which is consistent with earlier reports describing female sex as a predictor of simulator sickness (51). However, women also demonstrated a significantly greater increase in self-rated competence. Interestingly, male participants reported higher competence ratings before training, whereas post-training competence ratings were comparable between genders. The greater competence gain observed among female participants may therefore partly reflect lower baseline self-assessments rather than a true difference in educational benefit. Importantly, VR-related symptoms were not associated with competence gain (Spearman’s *ρ* = 0.05, *p* = 0.395). Considering the overall low symptom burden observed in our cohort and the predominantly mild nature of reported symptoms, it is unlikely that VR-related discomfort substantially influenced the perceived educational benefit of the training.

The gamified elements appear to have contributed to high acceptance. Gamification in medical education increases learner engagement, motivation, and perceived competence when aligned with learning objectives (29). In resuscitation training, gamified approaches have been associated with more positive attitudes toward acting in cardiac arrest situations (30). A systematic review emphasised the need to examine the underlying causes of improved learning and to assess resource requirements for implementation (52). However, another study revealed that VR training can result in cost savings of over 80% per individual when compared to traditional CPR training (53).

The recent ILCOR CoSTRs and the 2025 ERC Guidelines for Resuscitation acknowledge the growing evidence supporting VR in CPR training and its potential to improve skill acquisition and retention, by suggesting to consider VR as an alternative or supplement to traditional educational methods (6, 27, 28). Our study aligns with these recommendations by positioning VR as a complementary tool for theoretical preparation rather than a replacement for hands-on training. Our focus on gamification aligns with ILCOR’s recognition that current recommendations favour gamified learning in resuscitation (6, 52).

The successful integration into the OSCE preparation demonstrates the feasibility of incorporating immersive technologies into existing medical curricula. Students’ preferred settings included university training rooms (59%) and the integration into mandatory curriculum components (39%), suggesting VR training is most valued when embedded within structured educational contexts.

Traditional CPR training methods demonstrate poor skill retention after 6 months (8), while low-dose, high-frequency training programs show improved retention (9, 11). The accessibility and scalability of VR-based training may facilitate such repeated practice opportunities. Importantly, the high willingness to participate again (92%) training supports the LDHF paradigm recommended for CPR skill retention (9–12): intrinsically motivated learners are more likely to engage in the repeated, distributed practice that drives long-term competence.

Participants with prior VR experience reported significant higher usability and self-assessed performance ratings, highlighting the value of brief introductory sessions for VR-naive learners.

Trainers provided valuable implementation insights. The relatively short preparation time of about 10 min per each half-day session seems to be manageable even in busy clinical environments with teaching obligations. Trainers were able to supervise 4–6 students simultaneously in 60% of sessions, which is in line with other reports about reasonable supervision ratios (54). However, technical challenges were common: minor technical issues (1–3 times per session) occurred in 60% of sessions, headset handling required assistance in 79%, and navigation support within the VR scenario was needed in 53% of sessions. These findings underline the necessity of adequately trained personnel to provide technical support when implementing VR-based teaching.

### 4.1 Limitations

This study has several limitations. The observational single-centre design limits generalizability, and the absence of a control group prevents conclusions about relative effectiveness compared to traditional teaching methods. Although this study included a comparatively large cohort for an exploratory educational VR study, no formal *a priori* sample size calculation was performed. As the study was designed as an exploratory feasibility study focusing on usability and user experience, the sample size was determined pragmatically based on participant availability during the predefined study period. Therefore, the findings should be interpreted as exploratory and hypothesis-generating. Furthermore, the timing of VR training relative to other educational activities during the OSCE preparation period was neither standardized nor documented and may therefore have influenced study outcomes. All educational outcomes were based on self-assessment, without objective evaluation of rhythm analysis performance. A correlation between VR training participation and OSCE scores as an objective measurement was not possible due to the university’s privacy restrictions on exam results, which represents a critical validation gap. Additionally, neither the selected difficulty levels nor individual scenario performance were recorded by the VR application and were therefore unavailable for analysis. As participants were free to select, switch between, and repeat difficulty levels during training, differences in challenge level may have influenced study outcomes. Our findings are specific to the particular VR application evaluated and cannot be extrapolated to other VR-based resuscitation training tools. Although no participant was excluded because of motion sickness or epilepsy, self-selection bias cannot be excluded, as students concerned about VR-related adverse effects may have been less likely to volunteer for participation. The voluntary participation of the students introduces possible self-selection bias.

The study assessed only immediate post-intervention outcomes without measuring long-term retention, which would provide valuable insights into the durability of learning gains and is a knowledge gap. Finally, the study did not assess the impact on actual patient outcomes or clinical performance in real cardiac arrest situations. While training of medical personnel is associated with improved outcomes after cardiac arrest, establishing a direct causal link between VR training and patient outcomes would require large-scale, long-term studies with clinical endpoints.

This leads to future directions as randomized controlled trials comparing VR-based gamified rhythm analysis training to traditional instructions are needed to establish comparative effectiveness, incorporating objective assessments, and long-term follow-up. Research examining optimal integration within medical curricula, investigating the mechanisms underlying the effectiveness of gamified VR training, and developing strategies to minimise VR-induced symptoms, as well as cost effectiveness studies and possible impacts on health-related patient outcomes would advance the field.

### 4.2 Conclusion

In conclusion, this study suggests that VR-based, gamified rhythm-analysis training is a feasible and well-accepted adjunct to undergraduate resuscitation education, with high subjective usability ratings, minimal VR-related symptoms, and higher self-rated competence after training. In an exploratory subgroup-analysis women experienced more VR-induced symptoms but reported greater learning gains. Overall, the findings are consistent with current resuscitation education recommendations supporting VR as a promising supplement to blended CPR training rather than a replacement for traditional hands-on resuscitation training.

## Data Availability

The raw data supporting the conclusions of this article will be made available by the authors, without undue reservation.
