# Supplementary material for: Virtual reality, real-world rhythm: evaluating gamified training for medical students
Source: Front Public Health. 2026 Aug 3;14:1833908. doi: 10.3389/fpubh.2026.1833908 (PMC13479972; doi:10.3389/fpubh.2026.1833908)
Supplement: Supplementary file 1 [file Data_sheet_1.docx]

Questionnaire – Students

| Study ID: _ _ _ _ _ | Date: ______________________ | | | |
| --- | --- | --- | --- | --- |
| Example: J**o**n D**o**e, 3**1**.1**2**., V**i**enna  second letter first name, last name second digit day of birth, month of birth  second letter native town |  | | | |
|  | | - Morning | - Afternoon |  |

1. Gender

| - female | - divers | - open |
| --- | --- | --- |
| - male | - inter | - not specified |

2. Age

| - 21 - 23 | - 24 - 26 | - 27 - 29 |
| --- | --- | --- |
| - 30 - 32 | - 33 - 35 | - 36 - 38 |
| - 39 - 41 | - >42 |  |

3. Have you completed any professional medical training program?

| - Paramedic | - Registered Nurse | - None |
| --- | --- | --- |
|  | - other: ……………. |  |

4. Have you completed an accredited Advanced Life Support (ALS) course outside of a university program within the past two years (e.g., ERC, AHA)?

| - Yes | If yes, where and when: ________________________________ |
| --- | --- |
| - No |  |

5. Have you any prior experience with Virtual Reality (VR)?

| - Yes |  |
| --- | --- |
| - No |  |

6. How would you rate your own skill in performing a rhythm analysis **before** this VR-training?

| 1 |  |  |  | 5 |  |  |  |  | 10 |
| --- | --- | --- | --- | --- | --- | --- | --- | --- | --- |

1 – very poor

10 – excellent

7. How would you rate your own skill in performing a rhythm analysis **after** this VR-training?

| 1 |  |  |  | 5 |  |  |  |  | 10 |
| --- | --- | --- | --- | --- | --- | --- | --- | --- | --- |

1 – very poor

10 – excellent

8. How would you rate your performance in taking the appropriate actions based on the ECG rhythm displayed during the VR-training?

| 1 |  |  |  | 5 |  |  |  |  | 10 |
| --- | --- | --- | --- | --- | --- | --- | --- | --- | --- |

1 – very poor

10 – excellent

9. If you had to choose which training to complete, which would you prefer?

| - Virtual Reality Training | - Conventional Training |
| --- | --- |

10. Would you attend this VR-training again to practise rhythm analysis?

| - Yes | - No |
| --- | --- |

11. What aspects of this VR training session could be improved?

(Please provide all suggestions in the blank space below - Optional)

12. What aspects of traditional rhythm analysis teaching could be improved?

(Please provide all suggestions in the blank space below - Optional)

13. In what setting would you prefer to attend future training sessions?

| - as part of a compulsory university course | - as part of an elective subject |
| --- | --- |
| - open-access practice   rooms | - at home |

14. How would you rate the overall quality of this VR-training?

| 1 |  |  |  | 5 |  |  |  |  | 10 |
| --- | --- | --- | --- | --- | --- | --- | --- | --- | --- |

1 – very poor

10 – excellent

Virtual Reality System Usability Questionnaire (VRSUQ)

Evaluation of User Experience

*Mark how much each symptom below is affecting you right now with an* ***X***

|  |  | **1**  **Strongly disagree** | **2** | **3** | **4** | **5**  **Strongly agree** |
| --- | --- | --- | --- | --- | --- | --- |
| **1** | The system responded well to my manipulations as expected with no delays. |  |  |  |  |  |
| **2** | I think the virtual reality system provides clear feedback on my manipulations. |  |  |  |  |  |
| **3** | I kept making errors/mistakes while using the virtual reality system. |  |  |  |  |  |
| **4** | I could clearly understand the information presented within the virtual environment. |  |  |  |  |  |
| **5** | I think this system is user-friendly, straightforward to learn, and designed in such a way that most people will find it easy to adapt to. |  |  |  |  |  |
| **6** | I think it is easy to correct errors made during virtual reality experiences. |  |  |  |  |  |
| **7** | I enjoyed the virtual reality experience. |  |  |  |  |  |
| **8** | I felt dizzy, motion sickness, or a headache while experiencing virtual reality. |  |  |  |  |  |
| **9** | While experiencing virtual reality, I felt mental burdens such as tension, frustration, and time pressure. |  |  |  |  |  |

Items are rated on a 5-point Likert scale from 1 (strongly disagree) to 5 (strongly agree). Negatively worded items (errors, cybersickness symptoms, and mental stress) are reverse-coded so that higher values indicate better usability. The overall usability score is calculated as the mean of all items and transformed to a 0–100 scale using the formula (mean − 1) × 25, with higher scores representing greater perceived usability, as originally validated.

Virtual Reality Sickness Questionnaire (VRSQ)

*Mark how much each symptom below is affecting you right now with an* ***X***

|  |  | **1**  **None** | **2**  **Slight** | **3**  **Moderate** | **4**  **Severe** |
| --- | --- | --- | --- | --- | --- |
| **1** | **General discomfort** |  |  |  |  |
| **2** | **Fatigue** |  |  |  |  |
| **3** | **Headache** |  |  |  |  |
| **4** | **Eye strain** |  |  |  |  |
| **5** | **Difficulty focusing** |  |  |  |  |
| **6** | **Fullness of the head** |  |  |  |  |
| **7** | **Blurred vision** |  |  |  |  |
| **8** | **Dizziness with eyes closed** |  |  |  |  |
| **9** | **Vertigo** |  |  |  |  |

Thank you for your cooperation and for completing this questionnaire. We will provide information regarding the results in due course.
