# Supplementary material for: Virtual reality, real-world rhythm: evaluating gamified training for medical students
Source: Front Public Health. 2026 Aug 3;14:1833908. doi: 10.3389/fpubh.2026.1833908 (PMC13479972; doi:10.3389/fpubh.2026.1833908)
Supplement: Supplementary file 2 [file Data_sheet_2.docx]

Questionnaire - Trainer

*Please fill out this questionnaire after completing the five-hour training session*

| Trainer ID: ___________________  Example: J**o**n D**o**e, 3**1**.1**2**., V**i**enna  second letter first name, last name second digit day of birth, month of birth  second letter native town | | Date: _________________________ | | |
| --- | --- | --- | --- | --- |
|  | - Morning | | - Afternoon |  |

1. How much time did you spend to prepare the VR-glasses, student questionnaires and the training room?

_____ minutes

2. How many students did you have to supervise simultaneously?

(e.g.: training, explanation, technical assistance, discussion)

| - 1-3 | - 4-6 | - 7-9 | - >10 |
| --- | --- | --- | --- |

3. How stressful did you find the supervision of the students?

| 1 |  |  |  | 5 |  |  |  |  | 10 |
| --- | --- | --- | --- | --- | --- | --- | --- | --- | --- |

10 – very stressful

1 – not stressful

4. How often did technical issues occur during the five-hour training session?

| - 1-3 | - 4-6 | - 7-9 | - >10 |
| --- | --- | --- | --- |

5. Which technical issues did occur during that time?

(Please provide all suggestions in the blank space below)

6. How often did students require assistance with the VR-scenario during the five-hour training session?

| - 1-3 | - 4-6 | - 7-9 | - >10 |
| --- | --- | --- | --- |

7. How often did students require assistance using the VR-glasses during the five-hour training session?

| - 1-3 | - 4-6 | - 7-9 | - >10 |
| --- | --- | --- | --- |

8. What kind of assistance did students require with the VR-scenario?
   (Please provide all suggestions in the blank space below)
9. What kind of assistance did students require with the VR-glasses?
   (Please provide all suggestions in the blank space below)
10. How well did the students operate the glasses?

| 1 |  |  |  | 5 |  |  |  |  | 10 |
| --- | --- | --- | --- | --- | --- | --- | --- | --- | --- |

10 – excellent

1 – very poor

11. How high would you rate students` motivation during this VR-training?

| 1 |  |  |  | 5 |  |  |  |  | 10 |
| --- | --- | --- | --- | --- | --- | --- | --- | --- | --- |

10 – very high

1 – very low

12. How high would you rate students` motivation during conventional rhythm analysis training?

| 1 |  |  |  | 5 |  |  |  |  | 10 |
| --- | --- | --- | --- | --- | --- | --- | --- | --- | --- |

10 – very high

1 – very low

13. How high would you rate the learning success with this VR-application?

| 1 |  |  |  | 5 |  |  |  |  | 10 |
| --- | --- | --- | --- | --- | --- | --- | --- | --- | --- |

10 – very high

1 – very low

14. How high would you rate the learning success with conventional rhythm analysis training?

| 1 |  |  |  | 5 |  |  |  |  | 10 |
| --- | --- | --- | --- | --- | --- | --- | --- | --- | --- |

10 – very high

1 – very low

15. How satisfied were you as a trainer with Virtual Reality as a learning method?

| 1 |  |  |  | 5 |  |  |  |  | 10 |
| --- | --- | --- | --- | --- | --- | --- | --- | --- | --- |

10 – very satisfied

1 – very unsatisfied

16. How satisfied were you as a trainer with conventional training for rhythm analysis?

| 1 |  |  |  | 5 |  |  |  |  | 10 |
| --- | --- | --- | --- | --- | --- | --- | --- | --- | --- |

10 – very satisfied

1 – very unsatisfied

Thank you for your cooperation and for completing this questionnaire. We will provide information regarding the results in due course.
