# Supplementary material for: Virtual reality, real-world rhythm: evaluating gamified training for medical students
Source: Front Public Health. 2026 Aug 3;14:1833908. doi: 10.3389/fpubh.2026.1833908 (PMC13479972; doi:10.3389/fpubh.2026.1833908)
Supplement: Supplementary file 3 [file Data_sheet_3.docx]

Appendix C

# 1. Supplementary Histograms of Questionnaire and Performance Outcomes

Histograms are presented to facilitate visual assessment of outcome distributions and to support the use of non-parametric statistical methods.

## 1.1. Supplementary Figure S1. Distribution of usability scores

### 1.1.1. Figure S1A. Overall distribution

### 1.1.2. Figure S1B. Distribution by sex

### 1.1.3. Figure S1C. Distribution by prior medical qualification

### 1.1.4. Figure S1D. Distribution by prior VR experience

## 1.2. Supplementary Figure S2. Distribution of VR-induced symptom scores

### 1.2.1. Figure S2A. Overall distribution

### 1.2.2. Figure S2B. Distribution by sex

### 1.2.3. Figure S2C. Distribution by prior medical qualification

### 1.2.4. Figure S2D. Distribution by prior VR experience

## 1.3. Supplementary Figure S3. Distribution of competence change scores

### 1.3.1. Figure S3A. Overall distribution

### 1.3.2. Figure S3B. Distribution by sex

### 1.3.3. Figure S3C. Distribution by prior medical qualification

### 1.3.4. Figure S3D. Distribution by prior VR experience

## 1.4. Supplementary Figure S4. Distribution of self-rated performance scores

### 1.4.1. Figure S4A. Overall distribution

### 1.4.2. Figure S4B. Distribution by sex

### 1.4.3. Figure S4C. Distribution by prior medical qualification

### 1.4.4. Figure S4D. Distribution by prior VR experience

## 1.5. Abbreviations

IQR denotes interquartile range; VRSQ, Virtual Reality Sickness Questionnaire; VRSUQ, Virtual Reality Simulation Usability Questionnaire; VR, virtual reality.

## 1.6. Figure Note

Histograms show the distribution of each outcome overall and stratified by sex, prior medical qualification, and prior VR experience.
